# Bath Salts: A Newly Recognized Cause of Acute Kidney Injury

**DOI:** 10.1155/2012/560854

**Published:** 2012-10-24

**Authors:** Jonathan McNeely, Samir Parikh, Christopher Valentine, Nabil Haddad, Ganesh Shidham, Brad Rovin, Lee Hebert, Anil Agarwal

**Affiliations:** Division of Nephrology, Department of Internal Medicine, The Ohio State University Medical Center, Columbus, OH 43210, USA

## Abstract

Bath salts are substance of abuse that are becoming more common and are difficult to recognize due to negative toxicology screening. Acute kidney injury due to bath salt use has not previously been described. We present the case of a previously healthy male who developed acute kidney injury and dialysis dependence after bath salt ingestion and insufflation. This was self-reported with negative toxicology screening. Clinical course was marked by severe hyperthermia, hyperkalemia, rhabdomyolysis, disseminated intravascular coagulation, oliguria, and sepsis. We discuss signs and symptoms, differential diagnoses, potential mechanisms of injury, management, and review of the literature related to bath salt toxicity.

## 1. Introduction

 Detection of novel toxins that cause acute kidney injury (AKI) can be a diagnostic challenge, particularly if the manifestations of the toxin mimic those of well-established mechanisms of AKI. An example of this is “bath salt” intoxication, which causes a syndrome that may closely resemble severe sepsis. We report a case of a young adult male who developed severe hyperthermia, rhabdomyolysis, hypotension, leukocytosis, and dialysis-dependent AKI after ingestion and insufflation of bath salts. Had he not reported the bath salts exposure, confirmed by his family, we would have had no clue to his diagnosis because bath salts are not detected by routine drug screens of the urine or blood. Bath salts are sold legally in many states and are widely available on the Internet [1]. Their properties as synthetic stimulants have led to their widespread abuse. Bath salts can now be added to a list of other substances of abuse associated with acute kidney injury [2]. 

## 2. Case Report

 A 29-year-old Caucasian male was observed wandering the streets, agitated and behaving erratically. Later, he was found unresponsive, with multiple skin abrasions. He was transported to a local emergency department where he reported ingestion and insufflation (i.e., “snorting”) of bath salts, subsequently confirmed by family members. His past medical history included hepatitis C, posttraumatic stress disorder, polysubstance abuse, and tobacco abuse. He denied taking over-the-counter medications or herbal supplements. He had no known medical allergies.

On presentation, his blood pressure was 91/52 mm of Hg, pulse rate 93 per minute, and core temperature 107 degrees Fahrenheit. He was confused and agitated with no gross motor deficit. Pupils were 3 millimeters in size, equal, with no scleral icterus or conjunctivitis. Cardiac exam revealed no jugular venous distention, tachycardia, or murmur. Peripheral pulses were weak. Lungs were clear to auscultation bilaterally. Abdomen was soft with normal bowel sounds and no hepatosplenomegaly. Extremities were without peripheral edema or cyanosis. 

Table 1 summarizes laboratory data at presentation. It showed evidence of severe rhabdomyolysis. Serologic studies confirmed presence of hepatitis C, but were otherwise unremarkable. Serum toxicology testing was negative, including ethyl alcohol, acetaminophen, and salicylates. Urine toxicology detected lorazepam and cotinine, but was negative for more than 80 other substances, including cannabinoids, cocaine, ecstasy, ephedrine, lysergide, methamphetamine, methylphenidate, oxycodone, and phencyclidine. 

 Computed tomography scan of head without intravenous contrast, magnetic resonance imaging of cervical spine, electroencephalogram, and chest and abdominal X-rays were all normal. Right upper quadrant ultrasonography revealed no focal hepatic lesions, a normal pancreatic duct, and the presence of a few calculi within the gallbladder lumen; an echogenic right kidney with prominent corticomedullary differentiation was noted. 

**Figure 1:**
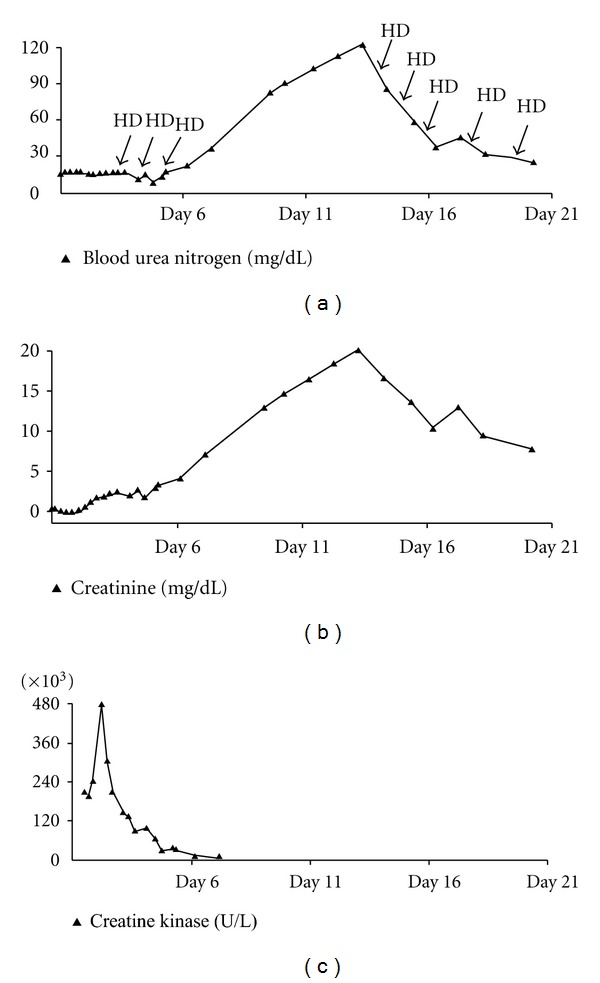
Blood urea nitrogen, creatinine, creatine kinase, and hemodialysis treatments. HD: intermittent hemodialysis treatment.

 He received intravenous fluids, required norepinephrine infusion for 18 hours, and received one 300 mg bolus of amiodarone. Hyperkalemia was treated with sodium polystyrene sulfonate, calcium gluconate, insulin, glucose and intravenous sodium bicarbonate. For severe hyperthermia he was given dantrolene and was placed under cooling blankets. Due to combativeness, he was sedated, intubated, and mechanically ventilated. Rasburicase was given for hyperuricemia. Urine output from an indwelling bladder catheter was 4,200 mL over the first 24 hrs, slowed to 825 mL over the next 24 hrs, and declined further to 150 mL per 24 hours for the next two days. He received hemodialysis daily three times, for both clearance and volume control. For persisting leukocytosis, all access lines were removed, and hemodialysis was held for 7 days; urine output remained negligible, serum creatinine steadily increased, and blood urea nitrogen (BUN) exceeded 100 mg/dL (Figure 1). With negative cultures, a tunneled dialysis catheter was placed for regularly scheduled hemodialysis treatments. Due to the patient's fulminant presentation and coagulopathy, a renal biopsy was not performed.

 For the first 8 days he had features of sepsis including disseminated intravascular coagulation (DIC), shock, lactic acidosis, liver failure, and non-ST elevation myocardial infarction. All peripheral blood cultures and urine cultures were negative. Electrocardiography demonstrated wide complex tachycardia.

He recovered, but remained dialysis dependent and was discharged to an extended care facility on the 22nd hospital day to receive regularly scheduled hemodialysis treatments which continued through at least 3 months of follow-up. At time of discharge, urine output remained <500 mL per day.

## 3. Discussion

 Bath salts are synthetic stimulants, sold in head shops and on the internet for up to $120 per gram [3]. Various preparations contain the stimulant designer drugs mephedrone (a.k.a. 4-methylmethcathinone) and MDPV (a.k.a. 3,4-methylenedioxypyrovalerone) [4, 5]. Mephedrone is classified as a beta-keto amphetamine [6, 7]. MDPV is structurally related to pyrovalerone, a monoamine reuptake inhibitor [8–14].

 Mephedrone exposure clinically manifests as agitation, anxiety, fever, chest pain, and palpitations [5, 13]. Distinctive signs and symptoms of MDPV exposure have yet to be published. Current drug screens in cases of suspected bath salt ingestion are often negative [5, 12]. Validated chromatographic methods for detecting mephedrone and MDPV have been published, but are not yet commonly available [7, 10]. Thus, making a definitive diagnosis of intoxication with these agents is difficult unless a diligent history is obtained. 

 Occurrence of severe rhabdomyolysis and dialysis-dependent AKI has not previously been reported with bath salts. We believe that rhabdomyolysis was the predominant cause of AKI in our patient and may have been related to direct muscle toxicity, severe hyperthermia, or electrolyte disorders. Amphetamines and amphetamine-like stimulants (e.g., MDMA) can cause rapidly progressive hyperthermia, cardiac arrhythmias, rhabdomyolysis, DIC, and multiorgan failure [15–18]. Renal failure is often attributable to myoglobinuria [19–21]. 

 The specific etiology of MDMA-induced hyperthermia is unclear [22, 23]. Amphetamines exhibit monoamine oxidase inhibitor (MAOI) activity, and direct activity at multiple receptors (e.g., 5-HT2, M1, H1, and *α*2-adrenergic) [20, 24]. MDMA exerts nonselective effects on postsynaptic serotonin (5-HT) receptors and inhibits 5-HT reuptake, increasing synaptic dopamine concentration. Dopamine by itself, or in conjunction with 5-HT, may be responsible for the hyperthermic effect of MDMA [25, 26]. MAOIs alter the metabolism of dopamine, norepinephrine, serotonin, and epinephrine, resulting in severe CNS excitation, and peripheral sympathetic stimulation [27–29]. MAOI toxicity is characterized by hyperpyrexia, hypotension, coma, rigidity, and seizures. Complications of MAOI overdose include AKI, asystole, coagulopathy, and rhabdomyolysis [30, 31]. Dantrolene has been used to treat MDMA-related hyperthermia [19, 20, 32].

 Could serotonin syndrome be responsible for our patient's rhabdomyolysis and AKI? Amphetamines and MDMA are known to cause serotonin syndrome, characterized by altered mental status, autonomic instability, and neuromuscular hyperactivity [17, 33]. Excessive muscle contraction may lead to hyperthermia and death. The risk of serotonin syndrome is increased with the combination of MAOIs and serotonin reuptake inhibitors (SRIs) [17, 34]. With negative urine screening for several common SRIs and other drugs with serotonergic activity, serotonin syndrome is less likely in our patient. However, manufacture of bath salts is unregulated and could contain other active substances that predispose to serotonin syndrome.

 Hyperthermia and multiorgan failure occur in neuroleptic malignant syndrome (NMS) and malignant hyperthermia (MH). NMS is associated with neuroleptic use and has a slow onset and progression (i.e., days) [25]. Our patient had a rapid progression and negative drug screening for many typical and atypical neuroleptics. MH presents within minutes to hours of exposure to volatile anesthetics and/or depolarizing muscle relaxants [25, 35, 36]. MH is an unlikely cause of our patient's AKI per se, as he had hyperthermia, hyperkalemia, and elevated creatine kinase prior to the administration of succinylcholine for intubation.

 Drug-induced fever occurs 7–10 days after starting a medication and is often limited to fever [37]. Oxidative phosphorylation uncoupling is less likely, given negative drug screening results for salicylates [38, 39]. Anticholinergic toxicity manifests with fever, tachycardia, psychosis, and seizures [35, 40–42]. Again, our patient's drug screen was negative for several such agents.

 Severe hyperkalemia was explained by acute renal failure and release of intracellular potassium associated with muscle injury. While arrhythmia is a noted complication in many of the above syndromes, the wide complex tachycardia seen in our patient was likely due to hyperkalemia [43, 44].

 Bath-salt-associated rhabdomyolysis had not been reported previously, although a similar case has appeared since the initial writing of this paper [45]. Treatment was based on usual treatment, including aggressive hydration and alkalinization. Whether early institution of hemodialysis would improve renal outcomes in severe rhabdomyolysis remains unproven. It is not known if hemodialysis—either continuous or intermittent—would help to remove the toxins associated with bath salts, although the nature of these compounds suggests a high volume of distribution and unlikelihood of effective removal.

 In summary, we report a case of bath salt use which resulted in severe hyperthermia, hyperkalemia, rhabdomyolysis, shock, DIC, and dialysis-dependent AKI with negative serum and urine drug screen for common toxins. We discuss possible mechanisms of AKI caused by toxic ingestion of bath salts. With increasing use of these substances of abuse and their easy availability, it is important to keep this possibility in differential diagnosis, especially when toxicologic screen is negative.

## Figures and Tables

**Table 1:** Laboratory Results.

Variable	Result upon presentation to emergency room	Result upon presentation to intensive care unit
Arterial blood gas		
pH	7.197	
pCO_2_ (mmHg)	58.7	
pO_2_ (mmHg)	527.9	
HCO_3_- (mmol/L)	22.3	
Serum chemistry (no hemolysis noted)		
Potassium (mmol/L)	8.1	4.7
Carbon dioxide (mmol/L)	16	19
Creatinine (mg/dL)	2.29	2.28
Blood urea nitrogen (mg/dL)	18	18
Calcium (mg/dL)	8.6	6.7
Albumin (g/dL)	4.4	3.7
Magnesium (mEq/L)		3.8
Phosphorous (mg/dL)		5.7
Uric acid (mg/dL)		21.2
Lactate (mg/dL)		3.4
Alanine aminotransferase (U/L)	81	535
Aspartate aminotransferase (U/L)	140	1169
Creatine kinase (U/L)	2771	201410
Myoglobin (ng/mL)	46780	
Troponin I (ng/mL)	0.541	3.92
Hematologic studies		
White blood cells (number ×1000/uL)		14.0
Hemoglobin (g/dL)		15.8
Platelets (number ×1000/uL)		150
Prothrombin time (sec)	15.2	27.7
International normalized Ratio	1.21	2.5
Partial thromboplastin time (sec)	30.6	53
Fibrinogen (mg/dL)		131
High sensitivity D-dimer (*μ*g/mL)		>20.00
Peripheral smear for schistocytes		Negative
Urinalysis		
Color	Red	
Blood	Large	
Casts (#/LPF)	>20 hyaline	
Red blood cells (#/HPF)	4	
